# Histopathological insights into mitral valve prolapse-induced fibrosis

**DOI:** 10.3389/fcvm.2023.1057986

**Published:** 2023-03-07

**Authors:** Maja-Theresa Dieterlen, Kristin Klaeske, Ricardo Spampinato, Mateo Marin-Cuartas, Karoline Wiesner, Jordan Morningstar, Russell A. Norris, Serguei Melnitchouk, Robert A. Levine, Antonia van Kampen, Michael A. Borger

**Affiliations:** ^1^ University Department of Cardiac Surgery, Heart Center Leipzig, HELIOS Clinic, Leipzig, Germany; ^2^ Department of Regenerative Medicine and Cell Biology, Medical University of South Carolina, Charleston, SC, United States; ^3^ Division of Cardiac Surgery, Massachusetts General Hospital, Harvard Medical School, Boston, MA, United States; ^4^ Cardiac Ultrasound Laboratory, Harvard Medical School, Massachusetts General Hospital, Boston, MA, United States

**Keywords:** mitral valve, fibrosis, mitral valve prolapse, histology, collagen

## Abstract

Mitral valve prolapse (MVP) is a cardiac valve disease that not only affects the mitral valve (MV), provoking mitral regurgitation, but also leads to maladaptive structural changes in the heart. Such structural changes include the formation of left ventricular (LV) regionalized fibrosis, especially affecting the papillary muscles and inferobasal LV wall. The occurrence of regional fibrosis in MVP patients is hypothesized to be a consequence of increased mechanical stress on the papillary muscles and surrounding myocardium during systole and altered mitral annular motion. These mechanisms appear to induce fibrosis in valve-linked regions, independent of volume-overload remodeling effects of mitral regurgitation. In clinical practice, quantification of myocardial fibrosis is performed with cardiovascular magnetic resonance (CMR) imaging, even though CMR has sensitivity limitations in detecting myocardial fibrosis, especially in detecting interstitial fibrosis. Regional LV fibrosis is clinically relevant because even in the absence of mitral regurgitation, it has been associated with ventricular arrhythmias and sudden cardiac death in MVP patients. Myocardial fibrosis may also be associated with LV dysfunction following MV surgery. The current article provides an overview of current histopathological studies investigating LV fibrosis and remodeling in MVP patients. In addition, we elucidate the ability of histopathological studies to quantify fibrotic remodeling in MVP and gain deeper understanding of the pathophysiological processes. Furthermore, molecular changes such as alterations in collagen expression in MVP patients are reviewed.

## Introduction

Mitral valve prolapse (MVP) is the most common heart valve disease with a prevalence of 2%–3% (1, 2). Myxomatous degeneration of the mitral valve (MV) leaflets eventually leads to systolic displacement of one or both mitral leaflets into the left atrium (3). The excessive mobility of the prolapsing leaflets along with increased systolic annular expansion is paralleled by superior papillary muscle (PM) displacement and a systolic curling motion of the inferobasal left ventricle (LV) wall (4–6). The structural link to the valve and potentially increased mechanical stress have previously been suggested as a possible mechanism for myocyte hypertrophy and fibrosis in these areas (7–10). According to this hypothetic mechanism, biomechanical stress might play a central role in the pathophysiology of the subvalvular and surrounding ventricular structural changes, i.e., replacement fibrosis, that occur secondary to MVP (11). Such fibrosis is clinically relevant because of a demonstrated association with ventricular arrhythmias and sudden cardiac death (SCD) (7, 9, 12). Cardiovascular imaging is the clinical noninvasive method used to detect myocardial fibrosis (13), while histological assessment is the validation standard for its detection and quantification (14, 15). To provide a clinical benefit for MVP patients in the future, three steps are recommended for preclinical and clinical research: (i) improvement and refinement in describing fibrosis, (ii) improvement in detecting fibrosis, and (iii) targeting fibrosis by surgical or pharmacological treatment (16). Therefore, the first step of analysis in the MVP patient cohort is the description of the quantity and quality of fibrosis and associated proteins to improve the knowledge about the involved pathological processes. The assessment of MVP patients using clinical, imaging and biological tools is necessary to evaluate the association to heterogeneous anatomic and pathophysiological development and progression of MVP (17).

This review provides an overview of current histopathological studies investigating fibrosis in LV myocardium in MVP patients.

## Myocardial fibrosis in MVP and fibrosis-related remodeling

In general, myocardial fibrosis is characterized by myofibroblasts expressing α-smooth muscle actin (α-SMA), formation of contractile polymerized stress fibers, and increased production of extracellular matrix (ECM) proteins such as collagen (14, 18, 19). Mechanical cardiac stress has been shown to induce specific molecular and biochemical alterations in the ECM by triggering an ECM-synthesizing program of structural and matricellular proteins (20). In addition, cardiac fibroblast proliferation is activated with increased expression of periostin which eventually leads to a transdifferentiation into myofibroblasts (1, 18, 19, 21). Whether these processes play a role in fibrosis development in MVP, as well as the underlying potentially biomechanical triggers have to be investigated in experimental mechanistic studies.

**Figure 1:**
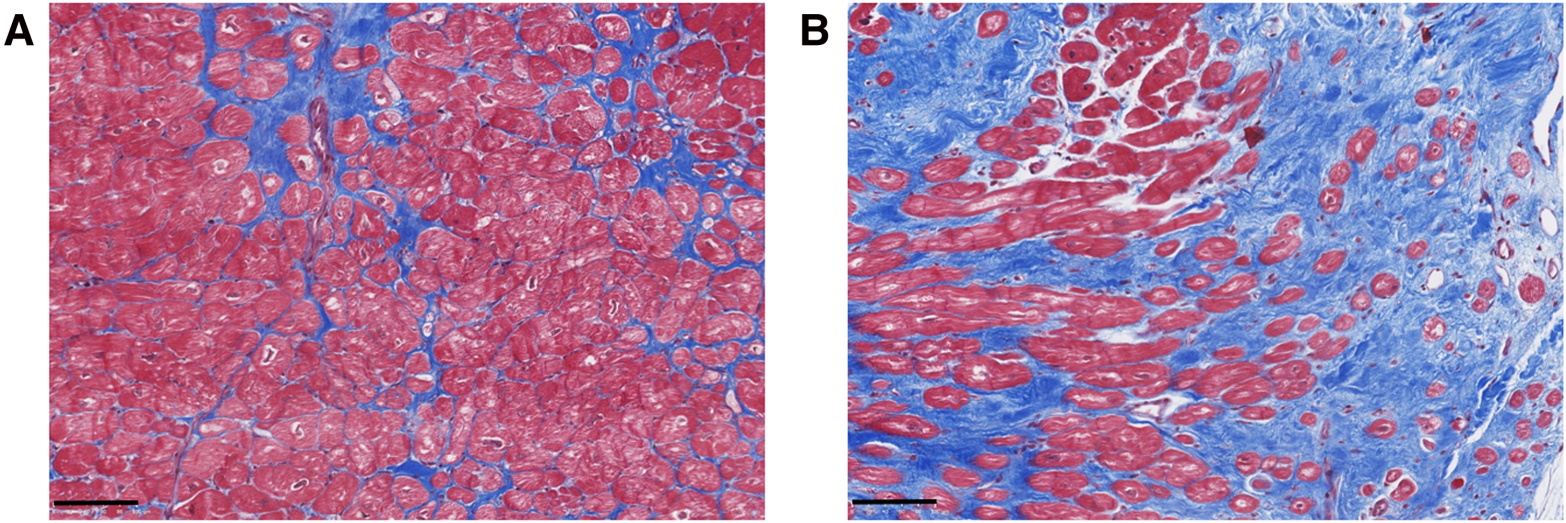
Exemplary histological analyses of Masson's trichrome stained cardiac biopsies of the inferobasal region of the left ventricular wall in MVP patients undergoing mitral valve repair surgery. Advanced interstitial fibrosis (**A**) and replacement fibrosis (**B**) can be detected. Scalebars = 100 µm.

Myocardial fibrosis in MVP is characterized by different types and structural distributions of fibrosis (Figure 1). This implies that the interaction between the prolapsing, myxomatous mitral leaflets, the PMs and the LV may result in nonuniform remodeling. This hypothesis was originally generated following investigation of MVP patients who died from SCD (7).

Hinderer et al. (18) described three types of myocardial fibrosis: reactive interstitial fibrosis, infiltrative interstitial fibrosis and replacement fibrosis. Reactive interstitial and replacement fibrosis have been found in MVP patients.

Reactive interstitial fibrosis is defined as an increase in ECM deposition without significant loss of cardiomyocytes (18). This type of fibrosis includes the excess of fibrous tissue in relation to cardiomyocytes within the myocardial interstitium, expanding the interstitial space in the form of microscars or bands of fibrous deposits surrounding cardiac muscle bundles or single cardiomyocytes (20). Moreover, fibrous deposits can be formed in the perivascular space around small blood vessels (20). In MVP patients, interstitial fibrosis has been found by CMR T1 mapping (22) and by histopathological investigations (23, 24).

Replacement fibrosis is a locally restricted, irreversible type of fibrosis forming a scar following cell necrosis (14, 18). In MVP specifically, replacement fibrosis has been found in the inferobasal myocardium and the PMs of patients with severe mitral regurgitation (MR) (3, 12, 23, 25), as well as in the LV myocardium at the level of the PMs and the adjacent free wall of patients who suffered SCD (12). Myocardial fibrosis in MVP patients can be characterized as interstitial, compact, focal, diffuse and patchy (3, 12, 18). In addition to focal cellular alterations and fibrosis within the PMs and LV wall, mitral leaflet fibrosis has also been detected in MVP (12, 23, 25). Based on the focal localization of fibrosis even in patients with mild MR, it has been hypothesized that its development occurs secondary to local traction of the myocardium by the prolapsing leaflets (12), a hypothesis that needs to be proved experimentally.

In addition to the reduction of the oxygen supply and nourishment in the myocardium, fibrosis causes electrical changes that predispose patients to arrhythmias (14). Diffuse interstitial fibrosis has been associated with ventricular arrhythmia in MVP patients and may be related to volume overload secondary to MR (22). On the other hand, localized fibrosis in myocardium adjacent to the PMs has been associated with ventricular arrhythmias and SCD in the absence of diffuse ventricular fibrosis (7, 9, 12). This supports the concept of mechanical stress from prolapsing valve structures causing localized fibrosis and subsequent arrhythmia substrate.

Determination of the different patterns of fibrosis and the optimization of the imaging techniques for better detection of these patterns is necessary, because the detection of different types of fibrosis could result in different clinical implications in the future. For example, it is known to date that irreversible replacement fibrosis is of prognostic relevance, and diffuse, interstitial fibrosis might be a dynamic, early and reversible marker of myocardial diseases (26).

Further, the pattern of fibrosis could be influenced by myocardial infarction, cardiomyopathy or genetic predispositions of MVP patients (26, 27). For example, interstitial fibrosis is mostly caused by chronic triggers such as pressure overload of inflammation, replacement fibrosis could occur after acute ischemic injury (28).

## CMR-based quantification of fibrosis in MVP patients

Noninvasive imaging plays a key role in direct or indirect detection of myocardial fibrosis with the use of multiple imaging modalities (29, 30). CMR remains the method of choice to detect myocardial fibrosis in daily practice (14, 31). The present review will provide a short overview about CMR-based technical opportunities and limitations of myocardial fibrosis detection compared to histology.

Late gadolinium enhancement (LGE)-CMR has a high sensitivity, specificity and reproducibility to assess focal myocardial replacement fibrosis by a relative accumulation of gadolinium in the extracellular matrix, but is not sensitive for identifying reactive interstitial fibrosis (13, 32–34). Furthermore, CMR T1 mapping is suitable to quantify diffuse interstitial fibrosis by increased extracellular volume (ECV) expansion and seems useful to detect an early stage of fibrosis (13, 31, 35). Additionally, higher native T1 times reflect myocardial disease involving the myocyte and interstitium and can be performed without the use of gadolinium-based contrast agents, and therefore without risk of renal dysfunction (14, 36).

Both CMR-derived LGE and T1 mapping sequences have been used to investigate myocardial fibrosis in MVP patients (11). LV replacement fibrosis detected by LGE-CMR was found in 36.7% of MR patients with MVP compared to 6.7% in MR patients without MVP, suggesting a unique pathophysiology independent of volume overload in MVP (37). Replacement fibrosis worsened with the degree of MR ranging from 13% in trace-mild MR to 37% in severe MR (38) and increased with patient age (37). The main pathologic findings in MVP patients are the focal fibrosis localized to the PMs and the LV wall adjacent to the PMs, including the inferolateral LV wall with an intramyocardial pattern (3, 12, 38). In addition, subendocardial patterns have been described (37).

In addition to focal and replacement fibrotic changes detected by LGE-CMR, diffuse LV interstitial fibrosis has been quantified by T1 mapping CMR sequences using pre- and post-contrast T1 times, and the calculated ECV fraction. ECV is a surrogate marker of diffuse LV interstitial fibrosis and shows a good correlation with histology in patients with cardiomyopathies, heart failure or mixed heart valve diseases (14, 31, 39). ECV is associated with LV dilation in asymptomatic patients with moderate or severe primary degenerative MR (40). Bui et al. showed a significant correlation between post-contrast T1 times and arrhythmias, LV dilation and MR severity, respectively, in patients with MVP (22). Moreover, they noted that among MVP patients with ventricular arrhythmias, only 36% had LGE in LV myocardium, and a shorter post-contrast T1 time was the only predictor of arrhythmias. More recently, ECV fraction by CMR was associated with increased MR severity and the presence of symptoms, but with a similar pattern in MVP and non-MVP cohorts (37). One previous study in patients with chronic primary MR observed a higher biopsy-proven LV fibrosis burden when compared to controls, even before the onset of symptoms (41). No significant relationships were identified between biopsy and CMR T1 mapping or LGE, however, possibly due to the patchy nature of fibrosis and the small amount of biopsy tissue obtained *via* an epicardial approach by these investigators. Instead, ECV correlated with multiple CMR parameters of LV function and with exercise capacity in this study (41). Furthermore, negative results of T1 mapping do not exclude future ventricular arrhythmias in MVP patients (35).

Previous reports showed that MVP patients with LV LGE had less MR (8). It was hypothesized, that the traction forces from a prolapsed MV leaflet are transmitted to the PM and LV wall inducing myocardial fibrosis (10). The Jensen group used a novel vacuum-based ex vivo model to demonstrate that the total PM force varies linearly with the trans-mitral pressure (42). Theoretically, more severe MR increases the left atrial pressure, which leads to a decrease in trans-mitral pressure gradient, with consequently lower PM traction force. Thus, hypothetically, MVP patients with pronounced flail leaflet and higher regurgitation volume might have less PM stretch.

CMR detection of fibrosis presents several limitations. For example, T1 mapping sequences can be affected by several artifacts. In addition, T1 sampling is sometimes performed in the septum of a single short-axis slice based on the assumption that it is a representative selection, which is not the case for every cardiac pathology (43). A major disadvantage of conventional bright-blood LGE is the poor contrast between scar tissue and the blood pool. Hence, the scar burden can be substantially underestimated or even completely obscured (44), especially in cases with subendocardial patterns or PM fibrosis. Particularly in pathologies with heterogeneous fibrotic distribution such as MVP, a histology-based quantification and characterization of fibrosis could be advantageous.

In the past 15 years, CMR-derived dark-blood LGE methods were introduced aiming to increase the scar-to-blood contrast and improved scar conspicuity (44, 45). Recently, Van de Heyning et al. compared dark and bright-blood LGE and found that dark-blood LGE-CMR improved the detection of LGE at the level of the PMs in MVP patients (46).

Correlating these imaging techniques with histological data would improve the informative value of imaging techniques regarding quantification of fibrosis in MVP, and long-term data collected with more sensitive imaging tools could provide information about the progression of fibrosis over time without additional biopsy sampling.

## Histology-based quantification of fibrosis in MVP patients

Histopathological analysis of myocardial biopsies is generally the gold standard for fibrosis detection and characterization, while CMR represents the gold standard for non-invasive detection of myocardial fibrosis (14). While in clinical practice CMR is utilized regularly, details of myocardial fibrosis can be examined more reliably by histological analysis, making the two methods complimentary techniques (20). In contrast to CMR, histological analysis of cardiac biopsies can identify the type of fibrosis (interstitial or replacement fibrosis) as well as structural distribution (e.g., diffuse, compact, focal) within the tissue. Furthermore, histological abnormalities such as fibrous deposits in the perivascular space of arterioles that also occur in MVP patients can be detected (20, 47).

Moreover, histology-based analysis provides the possibility to quantify the total collagen content, and to discriminate between different collagen types. Thus, histopathological analysis of myocardial biopsies may yield extremely valuable information in the context of mechanistic investigation of MVP-associated myocardial fibrosis, potentially leading to discovery of novel treatment options. Histological detection of fibrosis can furthermore guide advances in chemistry that provide specificity for collagen in CMR and positron emission tomography (PET) probes, with the potential for improved noninvasive detection of fibrosis (48).

With regard to MVP, there still is scarce knowledge about the development of myocardial fibrosis and the progression of fibrotic changes. Five studies have reported histological findings of fibrosis within the myocardium of MVP patients (Table 1). Three reports included tissue analyses of a multicentric patient cohort (12, 23, 49), while another two summarized investigations from single center cohorts (3, 50). In these studies, histological assessment was performed using hematoxylin eosin staining to identify abnormal histological features within the LV. Connective tissue was stained according to van Gieson or with trichrome and picrosirius red reagents (Table 1). These stains allow quantification of fibrosis and evaluation of fibrotic distribution.

**Table 1:** Overview of studies investigating myocardial fibrosis in MVP patients.

References	Study population	Analyses	Main findings
Morningstar, 2021 (3)	*n* = 6 patients with severe MR secondary to MVP and indication for MV repair	- Masson's trichrome stain - collagen stain	- pronounced replacement fibrosis within the inferobasal myocardium with little evidence of fibrosis within the apex or interventricular septum - increased collagen I protein in fibrotic regions - myocytes bordering the fibrotic zone with changes in cell size and presence of disorganized sarcomeres - peripapillary zone with numerous activated myofibroblasts - increase of CD206^+^ cells in the papillary region compared to septum or apex
Han, 2021 (48)	*n* = 17 SCD patients with MVP *n* = 17 age-, gender- and BMI-matched controls with noncardiac causes of death	- picrosirius red stain	- endocardial-to-epicardial gradient of fibrosis with the highest amounts in the inner third - more interventricular septum fibrosis in MVP-SCD victims than in controls - fibrosis in the RV free-wall comparable between MVP-SCD victims and controls - comparable fibrosis deposition in the anterior wall and the interventricular septum - comparable fibrosis deposition in the lateral and the posterior wall
Han, 2020 (22)	*n* = 70 autopsy cases of SCD with isolated MVP *n* = 70 autopsy cases of noncardiac, motor vehicle accident death	- hematoxilin eosin stain - connective tissue stain (van Gieson, trichrome or picrosirius red)	- abnormal LV histological features in 79% of MVP cases - patterns of interstitial and/or perivascular fibrosis - 24% multisegment fibrosis, 29% focal fibrosis, 24% multisegment and focal fibrosis - 24% without fibrosis - 85% fibrosis involving the subendocardial-midmural layer - 15% transmural fibrosis
Garbi, 2018 (49)	*n* = 68 SCD patients with MVP as cause of death	- hematoxilin eosin stain	- fibrosis found in 81% of the cases (89% confined to the LV, 11% involving the right ventricle) - focal, interstitial fibrosis within the inner wall, involving particularly the inner subendocardium, trabeculae and PMs - fibrosis confluent within the inner third of the LV basal posterior wall, with extensive replacement fibrosis extending into the trabeculae and posteromedial PM - 18% with fibrosis within the lateral and anterior LV wall - 24% with midwall interventricular septum fibrosis
Basso, 2015 (12)	*n* = 43 SCD patients with MVP *n* = 15 age- and sex-matched controls with noncardiac causes of death	- hematoxilin eosin stain - connective tissue stain (van Gieson, trichrome or picrosirius red)	- LV myocardium with increased endomysial and patchy replacement-type fibrosis at the level of PMs and adjacent free wall in 100% of the patients and in the subendocardial-midmural layer in the inferobasal wall under the posterior MV leaflet in 88% of the patients - focal fibrosis was present even with only mild MR - mean fibrous tissue % area in MVP SCD victims is 30.5% at the level of PMs and 33.1% in the inferobasal wall myocardium - increased cardiomyocyte diameters and dysmorphic/dysmetric nuclei in the LV myocardium at the level of PMs and the inferobasal wall

BMI, body mass index; LV, left ventricle; MR, mitral regurgitation; MV, mitral valve; MVP, mitral valve prolapse; PM, papillary muscle; RV, right ventricle; SCD, sudden cardiac death.

Three of the 5 histopathological studies investigating myocardial fibrosis in MVP patients analyzed cardiac tissue from autopsy cases of SCD victims with MVP (12, 23, 50). The advantage of analyzing autopsy cases is the availability of the whole heart for histopathological analysis, which allows more reliable results from different regions of the heart or the whole ventricular wall. In a study including 70 autopsy cases of SCD with isolated MVP and 70 autopsy control cases, 29% of the MVP SCD patients showed focal fibrosis, 24% multisegment fibrosis in the subendocardial-midmural LV layer, and 24% multisegment fibrosis plus focal midmural fibrosis (23). In 85% of all cases with fibrotic abnormalities, fibrosis involved the subendocardial-midmural layer of the ventricle wall, while 15% showed transmural fibrosis (23). In another report on MVP patients deceased from SCD, the same authors found an endocardial-to-epicardial gradient of fibrosis in the myocardium of MVP patients, with the highest amounts in the inner third (49).

A consistent pathological feature of MVP is the regionalized fibrosis of the PMs, the LV myocardium at the level of PMs and in the inferobasal region reported for 75%–90% of the cases (3, 12, 23, 50). Basso et al. quantified the fibrotic tissue area in the LV of MVP autopsy cases as 30.5% at the level of PMs and 33.1% in the inferobasal wall (12). Additionally, endomysial and patchy replacement fibrosis at the level of the PMs and the adjacent free wall in all patients as well as in the subendocardial-midmural layer in the inferobasal wall in 88% of the patients was reported, and the authors described increased cardiomyocyte diameters and dysmorphic nuclei in fibrotic areas of the LV myocardium (12). For 24% of the cases, a midwall interventricular septum fibrosis was reported (50).

It is hypothesized that the high-quality histological results obtained in autopsy cases with MVP do not fully explain the pathological processes of living MVP patients. Hence, it is important to consider investigations in living MR patients with syndromic or non-syndromic MVP. Our group recently published a small study cohort of six patients with severe MR secondary to MVP undergoing MV repair surgery (3). In biopsies obtained intraoperatively from valve-linked myocardium, we found pronounced replacement fibrosis within the inferobasal myocardium as well as myocytic bordering of the fibrotic zone with changes in cell size and the presence of disorganized sarcomeres. These changes were not present in control regions such as the interventricular septum and the LV apex. Fibrotic changes in the chordae tendineae and the surrounding tissue have also been described by other groups (25, 51). Interestingly, a higher grade of interstitial fibrosis was reported in MVP patients with idiopathic ventricular tachycardia (24).

Assessment of perivascular fibrosis by histological evaluation is an additional option to evaluate the progression of fibrosis in cardiac tissue. Because perivascular fibrosis might reduce the oxygen supply and tissue nourishment (52), its assessment and correlation with disease progression should be investigated. In MVP patients, perivascular fibrosis has been reported (23), but has not been quantified yet.

Despite the obvious advantages of histological fibrosis quantification, it remains limited by the amount of tissue that is available for analysis as well as sampling errors. Tissue analysis, especially when performed in living MVP patients, is locally restricted and there is risk of obtaining a non-representative biopsy. This risk can be reduced with bioptating at defined positions according to a standardized protocol. While studies with cardiac tissue of SCD victims offers the opportunity to obtain multiple and/or transmural samples from the LV wall (12, 49), the sampling in living patients is limited. For example, Morningstar et al. Obtained LV biopsies from the inferobasal LV wall (3).

However, histological quantification of representative biopsies is the most precise method to detect myocardial fibrosis and allows a very detailed and specific analysis of molecular changes, e.g., by determining the content of different collagen types.

## Collagen expression in MVP patients

Collagens form the ground substance of connective tissue (53). Fibrillar collagen I and collagen III are the major collagens in the human cardiac muscle with a share of 85% and 11%, respectively (18, 20). In myocardial fibrosis, an alteration of the collagen I/collagen III ratio occurs, which differs according to the respective underlying pathology (14, 18). For example, an increase of the ratio due to an excess of collagen I was documented for elderly patients, and patients with aortic stenosis and hypertensive heart disease (20). In contrast, patients suffering from ischemic heart disease tend to show a decreased collagen I/collagen III ratio caused by excessive collagen III expression (20). In general, an increase of the collagen I/collagen III ratio is associated with an increase of myocardial stiffness, because type I collagen fibers have a higher stiffness than type III collagen fibers (14, 54).

In MVP patients, investigations of collagen content and distribution in the myocardium are rare. Our group recently documented increased collagen I content in fibrotic LV regions of MVP patients (3). However, we did not compare our MVP patients to a control group. Instead, patients served as their own control by comparing valve-linked myocardium to LV regions not affected by prolapsing MV structures such as the LV apex and interventricular septum.

**Figure 2:**
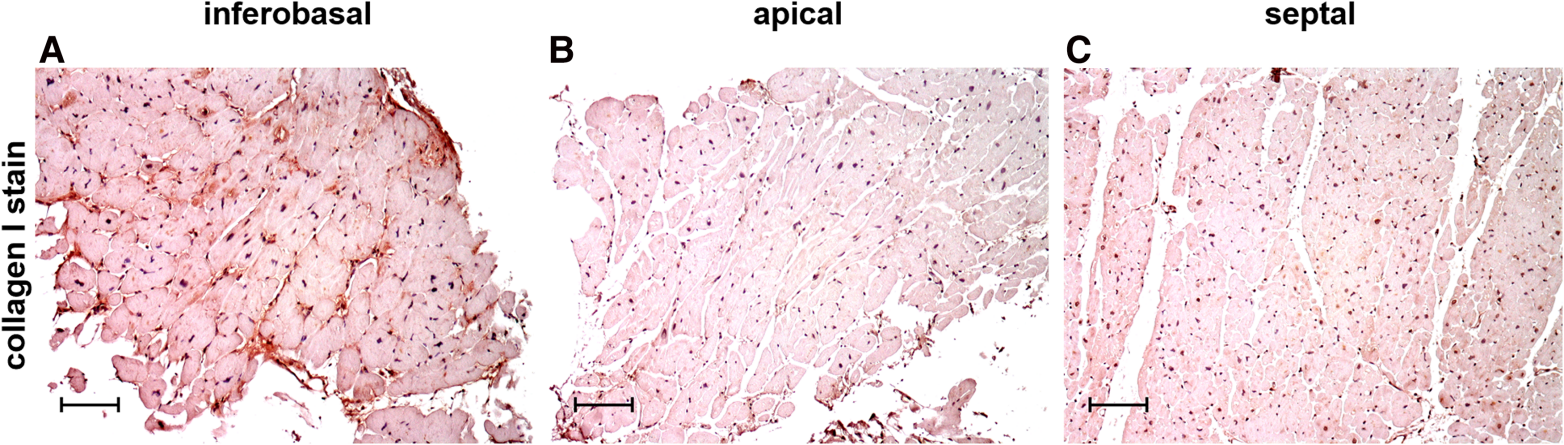
Immunohistochemical staining of collagen type I in cardiac biopsies obtained from the inferobasal (**A**), apical (**B**) and the septal regions (**C**) in MVP patients. Scalebars = 100 µm.

Figure 2 shows examples of immunohistochemical stains of collagen I and collagen III in exemplary LV regions of MVP patients, demonstrating that collagen content and distribution differ between regions. One major advantage of histological analysis is the ability to differentiate between different types of collagen. Immunohistochemical collagen I staining allows the differentiation of interstitial, compact, diffuse and patchy fibrotic structures (18). The length of collagen fibers gives further information on the type of fibrosis. While short collagen fibers are characteristic for diffuse fibrosis, longer fibers between myocardial bundles are commonly found in patchy fibrosis (18).

Cardiac collagen IV seems to play a role in myocardial fibrosis, because it induces the transdifferentiation of myofibroblasts and its disruption results in reduced fibrosis (18). With increasing remodeling activity, collagen deposition increases from the outer to the inner third of the LV free wall (20). The analysis of collagen expression in MVP cardiac tissue can help improve the understanding of disease extent and progression and may lead to novel mechanistic insights that can inform the development of therapeutic targets of myocardial fibrosis.

## Myocardial inflammation in MVP patients

Morningstar et al. described a regional LV inflammation in the peripapillary region of patients with MVP detected by CD206^+^ macrophage infiltration (3). Using hybrid PET-CMR, an association between degenerative, non-severe MR and occult myocardial inflammation was found that could explain that the majority of SCD victims with MVP do not have severe MR (55). These data point out that inflammatory processes could be involved in MVP-related myocardial fibrosis. The histological evaluation allows the further characterization and quantification of activated tissue-resident or infiltrated immune cells.

Monocytes, macrophages and mast cells are known to regulate cardiac fibrosis (56). For examples, infiltrated monocytes and macrophages are the primary source of fibrogenic growth factor amd cytokines, produce matricellular proteins and secrete matrix remodeling proteases (57), thereby exerting profibrotic effects in the myocardium. Myocardial resident mast cells mediate pro- and anti-fibrogenic signals be secreted proteins such as chymase and tryptase that induce TGF-β1 production (56) or vascular endothelial growth factor-A that increases capillary density in damaged cardiac tissue thereby inducing tissue repair (58, 59).

In addition, perivascular fibrosis, which has been reported in MVP patients (23), can be triggered by endothelial cells producing pro-inflammatory molecules that recruit lymphocytes and macrophages with fibrogenic potential (60).

The detection of infiltrated cells or perivascular fibrosis could give further hints to find the cause of LV fibrosis in MVP patients und underlines the necessity of histopathological investiagtions of the myocardium in the MVP population.

## Summary

In summary, the histopathological assessment of MVP-induced myocardial fibrosis in several studies has provided insight into the complex nature of fibrotic changes in MVP. To improve the clinical benefits in MVP patients in the future, correlating histological findings with imaging techniques, especially the different CMR methods such as bright- and dark-blood LGE, is necessary. Furthermore, the full spectrum of histological techniques such as staining with multiple dyes as well as immunohistochemistry for the detection of specific targets of interest should be used to further the understanding of disease development and progression. This includes the application of a classification for fibrosis type and distribution in MVP patients. The combination of advanced imaging methods and histological analyses may lead to novel mechanistic insights and possible therapeutic targets within the development of myocardial fibrosis in MVP.
